# *IL-1B* rs2853550 polymorphism contributes to esophageal cancer susceptibility in Chinese Han population of Northwest China

**DOI:** 10.1186/s10020-020-00178-y

**Published:** 2020-06-11

**Authors:** Ruimin Zhao, Xin Chen, Wanli Ren, Hao Dai, Huajing Li, Honghui Li, Ai Jia, Yue Wu, Peng Han, Yuan Shao

**Affiliations:** 1 Department of Otolaryngology & Head Neck, the First Hospital of Xi’an Jiaotong University, #227 West Yanta Road, Xi’an, 710061 Shaanxi Province China; 2 Department of Otolaryngology & Head Neck, Baoji Central Hospital, Baoji, 721008 Shaanxi China; 3 Department of Digestive System, The First Hospital of Xi’an Jiaotong University, Xi’an, 710061 Shaanxi China; 4 Department of Operation, the First Hospital of Xi’an Jiaotong University, Xi’an, 710061 Shaanxi China

**Keywords:** *IL-1B*, Polymorphisms, Esophageal cancer, Chinese Han population, Case-control study

## Abstract

**Background:**

Esophageal cancer (EC) is one of the most common human cancers, with a particularly aggressive behavior and increased incidence worldwide. The aim of this study was to assess the associations of single-nucleotide polymorphisms (SNPs) in *IL-1B* with the risk of EC in a northwest Chinese Han population.

**Methods:**

In order to evaluate the correlations between *IL-1B* polymorphisms and EC risk, an Agena MassARRAY platform was used to determine the genotype of the candidate SNPs among 384 EC patients and 499 controls. The associations between *IL-1B* variants and EC risk were examined using logistic regression analysis with adjustment for gender and age. Haplotype construction and analysis were performed to detect the potential associations between haplotypes within *IL-1B* and EC susceptibility. Additionally, bioinformatics databases were used for gene expression analysis and SNP functional prediction.

**Results:**

A significant relationship was found between *IL-1B* rs2853550 and an increased risk of EC in the allele model [odds ratio (OR) = 1.38, 95% confidence interval (95% CI): 1.01–1.89, *p* = 0.041), the codominant model (A/G, OR = 1.63, 95% CI: 1.10–2.42, *p* = 0.011), and the dominant model (OR = 1.49, 95% CI: 1.02–2.18, *p* = 0.041). Functional analysis revealed the potential effects of rs2853550, which further reinforced its influence on EC susceptibility. However, there were no statistically significant differences for other SNPs or haplotypes between EC cases and healthy controls. Expression analysis conducted with dataset indicated that the expression level of *IL-1B* was higher in EC cases than that in normal samples.

**Conclusions:**

This study demonstrated that rs2853550 in *IL-1B* might increase EC susceptibility in the Chinese Han population of Northwest China.

## Background

Esophageal cancer (EC) is the eighth most common human cancer, and its incidence rate has significantly increased in recent years (Parkin et al. 2005). It is noteworthy that during this time, most cases of EC have occurred in developing countries, and more than one-half of all EC cases were diagnosed in China, with EC being more common in males than in females, and more common in urban areas than in rural areas. Moreover, a previous study has reported that the 5-year survival rate of EC patients is extremely poor (< 20%) (Jemal et al. 2008). There are various habits that may contribute to esophageal tumorigenesis, two of these are alcohol consumption and cigarette smoking (Mayne et al. 2001). However, conclusive epidemiological studies have revealed that only a small proportion of individuals with such habits develop EC, which suggests that genetic aberrations, such as single-nucleotide polymorphisms (SNPs), are responsible factors that play important roles in EC development (An et al. 2015; Geng et al. 2015; Wang et al. 2016a; Wang et al. 2016b).

The IL-1 (interleukin-1) family exerts a pivotal effect on inflammatory and immune responses by affecting antigen recognition patterns and lymphocyte functions (Zheng et al. 2013). The *IL-1B* gene, which is located on human chromosome 2q13, belongs to a prototypical, multifunctional IL-1 gene cluster. The IL-1 beta (IL-1β) protein, encoded by *IL-1B*, functions as a secreted protein during both acute and chronic inflammation in the human body (Nicklin et al. 2002). IL-1β can be produced by different types of cells, such as T-lymphocytes, B-lymphocytes, natural killer cells, and neutrophils, but is primarily produced by blood monocytes and tissue macrophages (Bird et al. 2002). Previous studies have uncovered several significant *IL-1B* polymorphisms associated with diverse diseases in different cohorts worldwide. Majeed et al. have found a common polymorphic allele in the regulatory region of *IL-1B* that is associated with increased production of IL-1β in cervical lesions among European females (Majeed et al. 1999). *IL-1B* rs16944 has been demonstrated to affect *IL-1B* expression and was found to be associated with increased EC risk in an Irish population (Azim et al. 2004). Landvik et al. have found that individuals with the rs1143623 “C” allele of *IL-1B* have a reduced risk of lung cancer, with an odds ratio (OR) of 0.69 (95% CI: 0.51–0.92) (Landvik et al. 2008). Furthermore, Ito et al. have demonstrated that the *IL-1B* rs1143627 “A” allele plays a protective role in the development of breast cancer among postmenopausal women (Ito et al. 2002). Additionally, *IL-1B* variants have been proved to be associated with gastric cancer susceptibility (He et al. 2011a) and inflammatory bowel disease (Nemetz et al. 1999).

Although the significant evidence has be detected between *IL-1B* polymorphisms and multiple diseases in numerous populations. The relationships between *IL-1B* polymorphisms and EC susceptibility are seldom reported in the Han population from northwest China. Therefore, we performed the research that aimed to discover the potential roles of *IL-1B* variants in EC susceptibility. After reading the previous publications of *IL-1B* polymorphisms, we selected several SNPs as the candidate variations (Sasayama et al. 2011; Hovhannisyan et al. 2017; Kim et al. 2012; Langmia et al. 2016; Perez-Ramirez et al. 2017). Subsequently, these SNPs were searched in databases for detailed information and evaluated for primer design. In this study, we aimed to investigate the relationships between seven SNPs within *IL-1B*, namely rs2853550, rs1143643, rs3136558, rs1143630, rs1143627, rs16944, and rs1143623, and the risk of EC in a Han population from northwest China. Our results are supposed to provide significant evidence for the innate role of *IL-1B* and its polymorphisms in EC pathogenesis.

## Materials and methods

### Study subjects

This case-control study involved a Chinese Han population consisting of 384 EC patients (308 males and 76 females) and 499 controls (301 males and 198 females). All cases have been histopathologically diagnosed with EC and were consecutively enrolled from the Shaanxi Provincial People’s Hospital. It should be noted that patients who had undergone radiotherapy and chemotherapy or who had metastasized cancer were excluded from this study. Four hundred ninety nine control individuals were recruited from the physical examination center of the same hospital during the same period. None of the controls had personal or family cancer history, autoimmune, chronic or metabolic diseases. All the participants were permanent residents living in Shaanxi Province and had unrelated Chinese Han ancestries. Additionally, any patients or controls who recently received a blood transfusion were excluded from this study.

### Ethics approval and consent to participate

All participants were informed in writing and verbally of the procedures and purpose of this study. Signed informed consent documents were obtained from both patients and healthy individuals. Study protocols were approved by the Ethics Committee of the First Hospital of Xi’an Jiaotong University and Shaanxi Provincial People’s Hospital. Our research also complied with the ethical standards of the Ethics Committee and World Medical Association Declaration of Helsinki. All the subsequent research analyses were carried out in accordance with the approved guidelines and regulations.

### DNA isolation and SNP genotyping

Genomic DNA was isolated from peripheral blood samples using the GoldMag-Mini Purification kit (GoldMag Co. Ltd., Xi’an city, China), according to the manufacturer’s instructions. DNA concentrations were measured using a NanoDrop 2000 (Thermo Scientific, Waltham, Massachusetts, USA) at a wavelength of 260 nm. According to the previous publications, several polymorphisms in *IL-1B* were selected as the candidates (Sasayama et al. 2011; Hovhannisyan et al. 2017; Kim et al. 2012; Langmia et al. 2016; Perez-Ramirez et al. 2017). We searched these SNPs in 1000 Genomes database (http://www.internationalgenome.org/) (Fairley et al. 2019) and dbSNP database (https://www.ncbi.nlm.nih.gov/snp/) (Sherry et al. 2001) to screen the loci with minor allele frequencies (MAFs) > 0.05, and designed the primers with Agena Bioscience Assay Design Suite software, version 2.0 (https://agenacx.com/online-tools/) for amplification and single-base extension reactions (Gabriel et al. 2009). PCR primers for the seven SNPs are showed in Supplementary Table S1. The SNPs whose MAF <  0.05 and primers cannot be designed were excluded. *IL-1B* polymorphisms rs2853550, rs1143643, rs3136558, rs1143630, rs1143627, rs16944, and rs1143623, of which the MAFs were greater than 5% in the global population, were eventually eligible and genotyped in EC patients and healthy controls. SNP genotyping was conducted using the MassARRAY Nanodispenser and iPLEX platform (Agena Bioscience, San Diego, CA, USA), following the manufacturer’s protocol (Gabriel et al. 2009), and data were analyzed using Agena Bioscience TYPER software, version 4.0 (Gabriel et al. 2009; Thomas et al. 2007).

### Statistical analysis

SPSS 17.0 (SPSS, Chicago, IL, USA) were used for preliminary statistical analyses. Allele frequency of each SNP in the control group was calculated and Fisher’s exact test was performed to evaluate the departure from Hardy-Weinberg equilibrium (HWE). Variants with an HWE *p*-value greater than 0.05 were further analyzed. Fisher’s exact test and χ^2^ test were used to assess the differences in allele and genotype frequencies between patients and controls. The allele with low frequency was regarded as the minor allele “A”, and the other was the wild allele “B”. Furthermore, four genetic models (codominant: BB vs. AB vs. AA, dominant: BB vs. AB+AA, recessive: BB + AB vs. AA, and log-additive: for each A increase) were employed using SNPstats software (https://www.snpstats.net/start.htm) to estimate the relationship between each SNP and EC risk. Logistic regression analysis was carried out for association examinations, and odds ratio (OR) and 95% confidence intervals (95% CIs) were calculated using the logistic regression model (Bland and Altman 2000). As covariates, age and gender were adjusted to accurately assess statistical significance. Two-sided *p* values less than or equal to 0.05 were considered statistically significant for all statistical tests. Finally, Haploview software, version 4.2, and the SHEsis software platform (http://analysis.bio-x.cn/myAnalysis.php) (Yong and Lin 2005) were used for linkage disequilibrium (LD) assessments, and haplotype constructions and analyses.

### Bioinformatics analysis of *IL-1B* and functional assessments of SNPs

The public GEPIA database (Gene Expression Profiling Interactive Analysis; http://gepia.cancer-pku.cn/) (Tang et al. 2017) was used to analyze *IL-1B* expression differences between EC tumors and normal tissues. For SNP functional assessment, RegulomeDB (https://www.regulomedb.org/regulome-search/) (Boyle et al. 2012) and HaploReg (https://pubs.broadinstitute.org/mammals/haploreg/haploreg.php), version 4.1 (Ward and Kellis 2011), were used to predict the possible roles of the SNPs selected in this study.

## Results

### Population characteristics

The age and gender distributions of the 384 EC patients and 499 control subjects are listed in Table 1. The case and control groups were 80.3 and 60.3% male, respectively. The mean age [± standard deviation (SD)] of the case group was 60.81 ± 8.84 years at the time of diagnosis and that of the control group was 51.47 ± 11.84 years at recruitment. Statistical differences in age and gender existed between the two studied groups, however, were adjusted in subsequent analyses.

**Table 1:** Distribution of age and gender in EC patients and controls

Variable	Cases (n)	%	Controls (n)	%	*p* value
Total	384		499		
Gender					< 0.01^a^
Female	76	19.7	198	39.7	
Male	308	80.3	301	60.3	
Mean Age (years)	60.81		51.47		< 0.01^b^
Standard Deviation	8.84		11.84		

*p*^a^-value: *p*-value obtained using χ^2^ test

*p*^b^-value: *p*-value obtained from independent sample *t*-test

### *IL-1B* SNPs and EC risk

Basic characteristics and allele frequencies of *IL-1B* polymorphisms are presented in Table 2. SNP rs1143643 was excluded from this study because of its deviation from HWE (*p* <  0.05) in the control group. We evaluated the correlation between the *IL-1B* SNPs and EC susceptibility using four genetic models, hypothesizing that the minor allele of each variant was a risk factor. As showed in Table 2, the “A” allele of rs2853550 exhibited an obviously different frequency in patients compared with controls (11.7% vs. 8.8%) and was associated with an increased risk of EC (OR = 1.38, 95% CI: 1.01–1.89, *p* = 0.041). Genotype frequencies and risk association results of the selected *IL-1B* polymorphisms under the four genetic models are provided in Table 3. For variant rs2853550, the frequency of the heterozygous “A/G” genotype in patients was significantly different from that in controls (22.9% vs. 14.3%, respectively). Furthermore, compared with the “G/G” genotype in rs2853550, the “A/G” genotype contributed to an increased risk of EC after adjusting for age and gender (OR = 1.63, 95% CI: 1.10–2.42, *p* = 0.011). Additionally, after correction for age and gender, *IL-1B* rs2853550 was showed to be linked to an increased risk of EC based on the results of the dominant model (adjusted OR = 1.49, 95% CI:1.02–2.18, *p* = 0.041). However, *IL-1B* variants rs1143643, rs3136558, rs1143630, rs1143627, rs16944, and rs1143623 did not show evidence of a correlation with EC susceptibility in this cohort.

**Table 2:** Basic characteristics and allele frequencies of the seven *IL-1B* SNPs

SNP	Gene	Chromosome	Allele	Minor Allele Frequency		OR (95% CI)	*p*^a^
				Case	Control		
rs2853550	IL-1B	2q13	A < G	0.117	0.088	***1.38 (1.01–1.89)***	***0.041***
rs1143643	IL-1B	2q13	C < T	0.471	0.470	1.00 (0.83–1.22)	0.949
rs3136558	IL-1B	2q13	G < A	0.397	0.374	1.10 (0.90–1.33)	0.334
rs1143630	IL-1B	2q13	T < G	0.156	0.159	0.98 (0.76–1.27)	0.861
rs1143627	IL-1B	2q13	G < A	0.453	0.479	0.91 (0.75–1.09)	0.295
rs16944	IL-1B	2q13	A < G	0.456	0.474	0.93 (0.77–1.12)	0.444
rs1143623	IL-1B	2q13	G < C	0.362	0.399	0.85 (0.70–1.04)	0.114

*SNP* Single nucleotide polymorphism, *OR* Odds ratio, *95% CI* 95% confidence interval

*p*^a^ values were calculated using χ^2^ test for comparison of the allele distribution frequencies among EC patients and healthy controls

Bold italics indicates the SNP with statistical significance (*p* < 0.05)

**Table 3:** *IL-1B* SNPs and risk of EC with or without adjustment for gender and age

SNP	Genotypes	Controls, n (%)	Cases, n (%)	Without Adjustment		With Adjustment	
				OR (95% CI)	*p*^a^	OR (95% CI)	*p*^b^
rs2853550							
Codominant	G/G	417 (84.1%)	295 (76.8%)	1.00	***0.000***	1.00	***0.011***
	A/G	71 (14.3%)	88 (22.9%)	***1.75 (1.24–2.48)***		***1.63 (1.10–2.42)***	
	A/A	8 (1.6%)	1 (0.3%)	0.18 (0.02–1.42)		0.22 (0.03–1.86)	
Dominant (ref: G/G)	A/G + A/A	79 (15.9%)	89 (23.2%)	***1.59 (1.14–2.23)***	***0.007***	***1.49 (1.02–2.18)***	***0.041***
Recessive (ref: G/G + A/G)	A/A	8 (1.6%)	1 (0.3%)	0.16 (0.02–1.28)	0.032	0.20 (0.02–1.70)	0.082
Log-additive	–	–	–	***1.38 (1.01–1.89)***	***0.042***	1.30 (0.92–1.85)	0.140
rs3136558							
Codominant	A/A	197 (39.7%)	140 (36.6%)	1.00	0.620	1.00	0.980
	G/A	227 (45.8%)	181 (47.4%)	1.12 (0.84–1.50)		0.97 (0.70–1.35)	
	G/G	72 (14.5%)	61 (16.0%)	1.19 (0.80–1.79)		1.01 (0.64–1.59)	
Dominant (ref: A/A)	G/A + G/G	299 (60.3%)	242 (63.4%)	1.14 (0.87–1.50)	0.350	0.98 (0.72–1.34)	0.910
Recessive (ref: A/A + G/A)	G/G	72 (14.5%)	61 (16.0%)	1.12 (0.77–1.62)	0.550	1.02 (0.67–1.56)	0.920
Log-additive	–	–	–	1.10 (0.91–1.33)	0.340	1.00 (0.80–1.24)	0.980
rs1143630							
Codominant	G/G	352 (70.5%)	278 (72.4%)	1.00	0.360	1.00	0.220
	G/T	135 (27.1%)	92 (24.0%)	0.86 (0.63–1.17)		0.82 (0.58–1.17)	
	T/T	12 (2.4%)	14 (3.6%)	1.48 (0.67–3.24)		1.74 (0.72–4.17)	
Dominant (ref: G/G)	G/T + T/T	147 (29.5%)	106 (27.6%)	0.91 (0.68–1.23)	0.550	0.89 (0.64–1.25)	0.510
Recessive (ref: G/G + G/T)	T/T	12 (2.4%)	14 (3.6%)	1.54 (0.70–3.36)	0.280	1.83 (0.77–4.36)	0.170
Log-additive	–	–	–	0.98 (0.76–1.26)	0.860	0.98 (0.74–1.31)	0.910
rs1143627							
Codominant	A/A	124 (25.1%)	111 (30.6%)	1.00	0.170	1.00	0.580
	A/G	266 (54.0%)	175 (48.2%)	0.73 (0.53–1.01)		0.87 (0.61–1.25)	
	G/G	103 (20.9%)	77 (21.2%)	0.84 (0.56–1.23)		1.05 (0.68–1.64)	
Dominant (ref: A/A)	A/G + G/G	369 (74.8%)	252 (69.4%)	0.76 (0.56–1.03)	0.080	0.92 (0.65–1.30)	0.640
Recessive (ref: A/A + G/A)	G/G	103 (20.9%)	77 (21.2%)	1.02 (0.73–1.42)	0.910	1.15 (0.79–1.68)	0.470
Log-additive	–	–	–	0.90 (0.74–1.09)	0.290	1.01 (0.81–1.27)	0.900
rs16944							
Codominant	G/G	128 (25.6%)	115 (30.0%)	1.00	0.270	1.00	0.570
	G/A	269 (53.9%)	187 (48.8%)	0.77 (0.57–1.06)		0.90 (0.63–1.28)	
	A/A	102 (20.4%)	81 (21.1%)	0.88 (0.60–1.30)		1.11 (0.72–1.71)	
Dominant (ref: G/G)	G/A + A/A	371 (74.3%)	268 (70.0%)	0.80 (0.60–1.08)	0.150	0.96 (0.68–1.34)	0.790
Recessive (ref: G/G + G/A)	A/A	102 (20.4%)	81 (21.1%)	1.04 (0.75–1.45)	0.800	1.18 (0.82–1.72)	0.370
Log-additive	–	–	–	0.93 (0.76–1.12)	0.440	1.04 (0.84–1.29)	0.720
rs1143623							
Codominant	C/C	171 (34.5%)	154 (40.9%)	1.00	0.150	1.00	0.280
	C/G	254 (51.2%)	173 (45.9%)	0.76 (0.57–1.01)		0.79 (0.57–1.10)	
	G/G	71 (14.3%)	50 (13.3%)	0.78 (0.51–1.19)		1.05 (0.65–1.70)	
Dominant (ref: C/C)	C/G + G/G	325 (65.5%)	223 (59.1%)	0.76 (0.58–1.00)	0.054	0.84 (0.62–1.15)	0.280
Recessive (ref: C/C + C/G)	G/G	71 (14.3%)	50 (13.3%)	0.92 (0.62–1.35)	0.660	1.19 (0.76–1.87)	0.440
Log-additive	–	–	–	0.85 (0.70–1.04)	0.110	0.96 (0.76–1.20)	0.700

*SNP* Single nucleotide polymorphism, *OR* Odd ratio, *95% CI* 95% confidence interval, *Ref* Reference category

*p*^a^: *p*-values calculated by logistic regression analysis

*p*^b^: *p*-values calculated by logistic regression analysis with adjustments for gender and age

Bold italics indicates the SNP with statistical significance (*p* < 0.05)

### EC risk and *IL-1B* haplotypes on chromosome 2q13

**Fig. 1:**
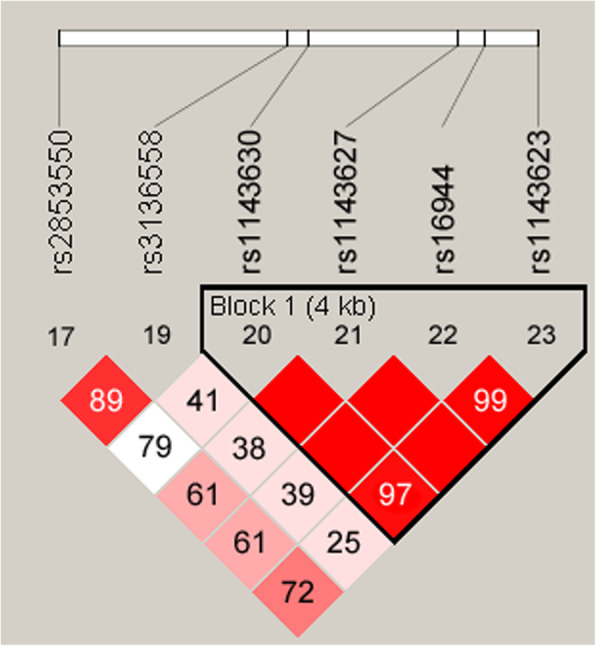
Illustration of the haplotype block. Four *IL-1B* polymorphisms (rs1143630-rs1143627-rs16944-rs1143623) mapped to a 4-kb LD block. The red squares in the *IL-1B* LD block indicate significant linkages between the four SNPs. The numbers in squares are the values of D’. Different numbers show the degree of LD of SNPs. The bright red squares with no number in the middle have the D’ of 100

Finally, four *IL-1B* polymorphisms (rs1143630, rs1143627, rs16944, and rs1143623) mapped to a 4-kb LD block, forming four haplotypes with frequencies greater than 0.05 among our subjects (Table 4). In Fig. 1, the red squares in the *IL-1B* LD block indicated significant linkages between the four SNPs. Unfortunately, there were no statistically significant correlations between any *IL-1B* haplotype and EC risk in our cohort (Table 4).

**Table 4:** Haplotype analysis of the *IL-1B* block formed by rs1143630, rs1143627, rs16944, and rs1143623, and the association with EC risk

Haplotype Block	Frequency (Case)	Frequency (Control)	Without Adjustment		With Adjustment	
			OR (95% CI)	*p*^a^	OR (95% CI)	*p*^b^
GAGC	0.54	0.52	1.00	–	1	–
GGAG	0.21	0.24	0.83 (0.65–1.06)	0.130	0.99 (0.74–1.31)	0.930
TGAG	0.15	0.16	0.94 (0.72–1.24)	0.680	1.00 (0.74–1.36)	0.990
GGAC	0.09	0.07	1.19 (0.83–1.71)	0.350	1.28 (0.85–1.93)	0.240

*OR* Odds ratio, *95% CI* 95% confidence interval

*p*^a^: *p*-values calculated by logistic regression analysis

*p*^b^: *p*-values calculated by logistic regression analysis with adjustments for gender and age

### Potential functional roles of the selected *IL-1B* SNPs

Using the RegulomeDB and HaploReg databases, we assessed the possible functions of the seven selected SNPs, and the results are listed in Supplementary Table S2. All variants were predicted to have biological functions, according to the combined annotations of the two online tools. Their functional importance, particularly those of rs2853550, rs1143627, and rs16944, was evident from the low RegulomeDB score and the prediction of multiple functions in HaploReg.

### Expression analysis of *IL-1B* gene in EC

**Fig. 2:**
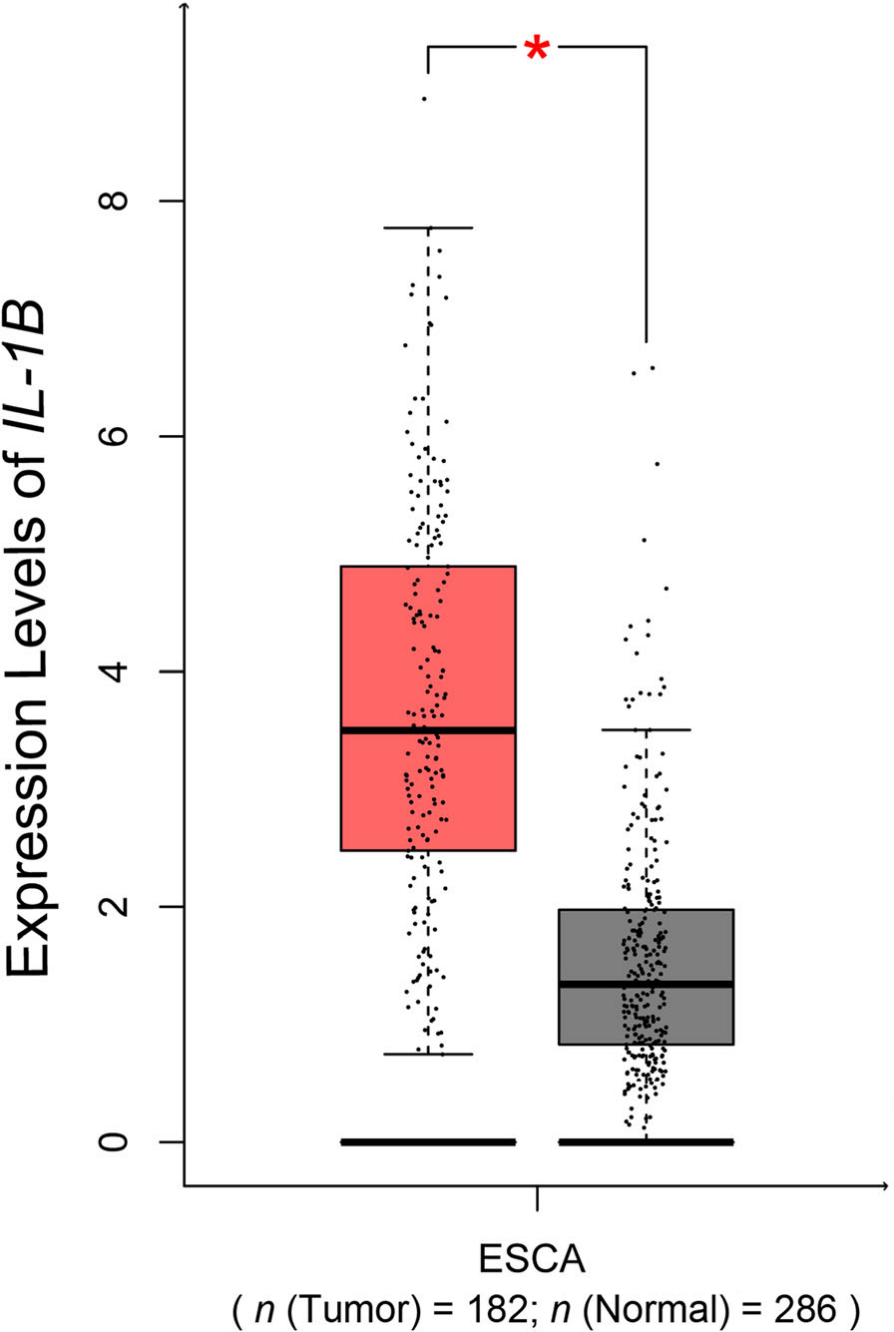
*IL-1B* expression patterns in EC tumors and normal tissues. *IL-1B* expression patterns were assessed using GEPIA (Gene Expression Profiling Interactive Analysis; http://gepia.cancer-pku.cn/) database. Expression of *IL-1B* is increased in EC tumors (*n* = 182) compared with normal tissues (*n* = 286). ESCA: Esophageal carcinoma

GEPIA analysis revealed a significant difference in *IL-1B* expression levels between 182 EC tumors and 286 normal tissues (Fig. 2). The expression level of *IL-1B* was increased in tumor samples when compared with controls (*p* <  0.001).

## Discussion

In the present study, we genotyped seven polymorphisms in *IL-1B* and systematically evaluated their correlations with EC risk in a Chinese Han population of Northwest China. Our data yielded statistical evidence of an association between the SNP rs2853550 and increased risk of EC for the first time. Furthermore, functional analysis showed the potential effect of rs2853550 polymorphism, which strengthens the prospect that rs2853550 contributes to EC susceptibility. Additionally, we evaluated the *IL-1B* expression pattern in EC and normal samples using convincing bioinformatics analysis. The expression level of *IL-1B* was up-regulated in EC tissues.

IL-1B, an IL-1 family protein, is an important mediator of inflammatory responses and is involved in cell proliferation and apoptosis during tumor development. The accumulation of IL-1B can assist the body in fighting infection by increasing the expression of adhesion factors on endothelial cells, leading to the migration of leukocytes to sites of infection and altering the set point of the hypothalamic thermoregulatory center, resulting in an elevated body temperature (He et al. 2011a). However, mounting evidence has showed that inflammatory responses are closely related to the development of cancer (Werb LMC, Zena 2002). The importance and influence of *IL-1B* in tumor development and tumor immunity have been conclusively demonstrated for various diseases (Zheng et al. 2013; Witkin 2002; Upadhyay et al. 2008). In this study, the expression of *IL-1B* was dramatically increased in EC tissues compared to normal samples from the GEPIA database, suggesting that *IL-1B* promotes malignancy. Therefore, we speculated that *IL-1B* acts as a stimulating factor during tumorigenesis. Moreover, it is biologically reasonable that functional *IL-1B* polymorphisms play potential roles in the development of EC.

The wide variation in EC incidence across populations worldwide might be influenced by differences in a group’s genetic predisposition. Polymorphisms within the inflammatory and immune factor-encoding *IL-1B* gene can result in aberrant IL-1β levels, and their effects on oncogenic processes have been validated in diverse diseases (Ozbabacan et al. 2014). The association of *IL-1B* rs2853550 with cancer risk has been discussed in several studies. He et al. have reported that *IL-1B* rs2853550 heterozygotes (OR = 0.34, 95% CI: 0.2–0.7, *p* = 0.0028) and the “A” allele are associated with a significantly reduced risk of colorectal cancer (OR = 0.43, 95% CI: 0.2–0.9, *p* = 0.0015) (He et al. 2011b). Two other independent studies have revealed that the “A” allele of *IL-1B* rs2853550 might reduce the risk of bowel disease and gastric cancer when was compared with the “G” allele (Nemetz et al. 1999; He et al. 2011b). Our study is the first to provide evidence of the relationship between the SNP rs2853550 and increased EC risk in a northwest Chinese Han cohort. The results showed that the “A/G” genotype of rs2853550 contributed to an increased EC risk compared with the “G/G” genotype. In accordance with database analyses, we hypothesize that the rs2853550 polymorphism might influence the expression of *IL-1B* and thereby affect individual susceptibility to EC. However, Ito and colleagues did not find any association of the *IL-1B* rs2853550 polymorphism with EC risk in Japanese people, which might be attributable to the limited sample size of the Japanese population in that study (75 EC patients and 136 controls) (Ito et al. 2007). Zheng et al. have showed evidence of an association between the *IL-1B* rs16944 G > A polymorphism and EC risk in a southern Chinese Han population using SNP association analyses (Zheng et al. 2013). Nevertheless, a correlation between rs16944 and EC risk could not be established in our northwest Chinese Han population, which underscores the importance of genetic heterogeneity in population-based research. Further, differences in these studies might also be due to regional disparities, varying sample sizes, and the complexity of genetic and environmental interactions in different ethnic groups. Additionally, we did not find any associations of rs1143643, rs3136558, rs1143630, rs1143627, and rs1143623 with esophageal cancer susceptibility in this work. Although the potential functional roles of these SNPs were provided by databases annotating potential predicted regulatory elements, individual variation does not exert their effects alone and their functions might be influenced by genetic backgrounds.

Several limitations in this study should be noted. First, our study found the significant evidence of rs2853550 in correlation to EC development in Chinese Han population of Northwest China. Studies with larger sample size, preferably of different ethnicties, need to be performed to confirm the significant role of rs2853550. Second, we performed a function prediction of rs2853550 with database, however, the biological function exerted by rs2853550 need to be further elucidated with well-designed studies. Therefore, further confirmation is necessary to better understand the role of *IL-1B* rs2853550 in EC risk.

## Conclusions

In summary, our study provides strong evidence that the *IL-1B* rs2853550 polymorphism could contribute to the risk of EC in the northwest Chinese Han population. These findings indicate a role for *IL-1B* during EC development and might provide new targets and strategies for EC risk assessment in the northwest Chinese Han population.

## Supplementary information

**Additional file 1 Supplementary Table S1.** Primers used for the identification of the *IL-1B* polymorphisms. **Supplementary Table S2**. Functional analysis of selected *IL-1B* SNPs based on two databases.

## Data Availability

The datasets used and analysed during the current study are available from the corresponding author on reasonable request.

## Abbreviations

EC: Esophageal cancer
ESCC: Esophageal squamous cell carcinoma
EAC: Esophageal adenocarcinoma
SNPs: Single-nucleotide polymorphisms
MAF: Minor allele frequency
HWE: Hardy-Weinberg equilibrium
OR: Odds risk
95%CI: 95% confidence interval
SD: Standard deviation
LD: Linkage disequilibrium
