# Supplementary material for: IL-1B rs2853550 polymorphism contributes to esophageal cancer susceptibility in Chinese Han population of Northwest China
Source: Mol Med. 2020 Jun 11;26:57. doi: 10.1186/s10020-020-00178-y (PMC7291710; doi:10.1186/s10020-020-00178-y)
Supplement: Supplementary file 1 — Additional file 1 Supplementary Table S1. Primers used for the identification of the IL-1B polymorphisms. Supplementary Table S2. Functional analysis of selected IL-1B SNPs based on two databases. [file 10020_2020_178_MOESM1_ESM.docx]

Supplementary Table S1 Primers used for the identification of the *IL-1B* polymorphisms

| SNP | First PCRP (5'–3') | Second PCRP (5'–3') | UEP (5'–3') |
| --- | --- | --- | --- |
| rs2853550 | ACGTTGGATGCGAAGACTATCCTCCTCACC | ACGTTGGATGTGCAGTGCTTCAGCTGATCC | CAGCTGATCCTGTTCCA |
| rs1143643 | ACGTTGGATGCCTCAGCATTTGGCACTAAG | ACGTTGGATGACTCCTGAGTTGTAACTGGG | GGGCCCCCAACTTTC |
| rs3136558 | ACGTTGGATGAAGGGCTTGAAAGAATCCCG | ACGTTGGATGGATTCATCCACCTCGGCTTC | AACCCGCCTGGCCCAGAGAGGGATGA |
| rs1143630 | ACGTTGGATGTCTTGAGTCTGCCTCTAACC | ACGTTGGATGAGATTATCCCTCTCTGAAGC | AGCTCAAGGAGGTTAAG |
| rs1143627 | ACGTTGGATGTCTCAGCCTCCTACTTCTGC | ACGTTGGATGTTGTGCCTCGAAGAGGTTTG | GTTCCCTCGCTGTTTTTAT |
| rs16944 | ACGTTGGATGCTGTCTGTATTGAGGGTGTG | ACGTTGGATGAGAGGCTCCTGCAATTGACA | AATTGACAGAGAGCTCC |
| rs1143623 | ACGTTGGATGACCTATTTCCCTCGTGTCTC | ACGTTGGATGATGTGCCAGGTATCGTGCTC | TTTAGTGCTCGCTCTGCATTAT |

**SNP: Single nucleotide polymorphism; PCRP: PCR primer; UEP: Unextended mini sequencing primer.**

Supplementary Table S2 Functional analysis of selected *IL-1B* SNPs based on two databases

| SNP | Chr: Position | Role | RegulomeDB Score^a^ | HaploReg |
| --- | --- | --- | --- | --- |
| rs2853550 | 2:113587121 | Downstream | 3a | Enhancer histone marks, DNAse, Proteins bound, Motifs changed |
| rs1143643 | 2:113588302 | Intron | 6 | DNAse, Motifs changed, Selected eQTL hits |
| rs3136558 | 2:113591275 | Intron | 5 | DNAse, Motifs changed |
| rs1143630 | 2:113591655 | Intron | No Data | DNAse, Motifs changed |
| rs1143627 | 2:113594387 | Promoter | 1b | DNAse, Proteins bound, Motifs changed, Selected eQTL hits |
| rs16944 | 2:113594867 | Promoter | 1f | DNAse, Motifs changed, Selected eQTL hits |
| rs1143623 | 2:113595829 | Promoter | No Data | DNAse, Motifs changed, Selected eQTL hits |

**SNP: Single nucleotide polymorphism; eQTL: Expression quantitative trait loci; Chr: chromosome.**

**^a^Predicted functional effects of each variant as scored by the RegulomeDB database.**
